# Dr. Hamilton Again

**Published:** 1896-12

**Authors:** 


DR. HAMILTON AGAIN: Criticising his action in resigning from the Marine Hospital service,—we, in our November issue, unwittingly placed Dr. Hamilton in a false light. It was because we were not in possession of all the facts. Our statements were based, however, upon the remarks of the medical press generally. We are now in receipt of information which is reliable and trustworthy, which goes to show that Dr. Hamilton was under no obligations whatever to the surgeon-general, and that the privilege he enjoyed of holding other positions while in charge of the Marine Hospital at Chicago, was not by reason of any favoritism on the part of the surgeon-general; but on the contrary, Dr. Wyman owes his promotion to the office of surgeon-general solely to Dr. Hamilton; and it is he, therefore, who has not shown a becoming appreciation of the situation.

Dr. Hamilton found the office of surgeon-general not remunerative, the salary being only $4000,—by reason of the expense attending his efforts to secure medical legislation, and the cost of living, in Washington; and when he was offered a chair in Rush Medical College, with a salary of $2000, he proposed to the President and the Secretary of the Treasury to take charge of the Marine Hospital at Chicago, retaining, of course, his rank as surgeon. The salary of the two positions, combined, was better pay; and there were other considerations. This was agreed to by the President, Mr. Harrison, and the Secretary, with the further agreement that the arrangement was to hold for two Presidential terms. Proof of this compact can be forthcoming, we are assured, if necessary. "Thereupon," says our informant, "Dr. Hamilton was induced to reccommend Dr. Walter Wyman—his assistant—for the vacancy," and Dr. Wyman received the appointment. Of course Dr. Wyman knew of the arrangements, and having profitted by it—he was in honor bound, it seems to us, to at least permit it to be consummated. His cornering Dr. Hamilton so as to force his resignation from the service altogether, looks like a species of ingratitude, if not downright meanness.

